# Biomarkers for the severity of periodontal disease in patients with obstructive sleep apnea:IL-1 β, IL-6, IL-17A, and IL-33

**DOI:** 10.1016/j.heliyon.2023.e14340

**Published:** 2023-03-14

**Authors:** Mayra A. Téllez Corral, Eddy Herrera Daza, Natalia Arango Jimenez, Darena Z. Morales Vera, Juliana Velosa Porras, Catalina Latorre Uriza, Francina M. Escobar Arregoces, Patricia Hidalgo Martinez, María E. Cortés, Liliana Otero, Claudia M. Parra Giraldo, Nelly S. Roa Molina

**Affiliations:** a Centro de Investigaciones Odontológicas, Faculty of Dentistry, Pontificia Universidad Javeriana, Bogotá, D.C., Colombia; b Department of Mathematics, Faculty of Sciences, Pontificia Universidad Javeriana, Bogotá, D.C., Colombia; c Periodontics, Faculty of Dentistry, Pontificia Universidad Javeriana, Bogotá, D.C., Colombia; d Sleep Clinic, Hospital Universitario San Ignacio and Faculty of Medicine, Pontificia Universidad Javeriana, Bogotá, D.C., Colombia; e Faculty of Dentistry and Innovation Technology Graduate Program, Universidade Federal de Minas Gerais, Belo Horizonte, Minas Gerais, Brazil; f Human Proteomics and Mycoses Laboratory, Faculty of Sciences, Pontificia Universidad Javeriana, Bogotá, D.C., Colombia

**Keywords:** Cytokines, Saliva, Gingival crevicular fluid, Periodontitis, Obstructive sleep apnea

## Abstract

**Objective:**

This study aims to compare the salivary and gingival crevicular fluid (GCF) concentrations of five cytokines: IL-1β, IL-6, IL-17A, IL-33, and Tumor Necrosis Factor-alpha (TNF-α) in patients with OSA and their association with periodontitis.

**Methods:**

Samples of saliva and GCF were obtained from 84 patients classified into four groups according to periodontal and OSA diagnosis: G1(H) healthy patients, G2(P) periodontitis and non-OSA patients, G3(OSA) OSA and non-periodontitis patients, and G4(P-OSA) periodontitis and OSA patients. The cytokines in the samples were quantified using multiplexed bead immunoassays. Data were analyzed with the Kruskal-Wallis test, Dunn's multiple comparisons test, and the Spearman correlation test.

**Results:**

Stage III periodontitis was the highest in patients with severe OSA (69%; *p=*0.0142). Similar levels of IL-1β and IL-6 in saliva were noted in G2(P) and G4(P-OSA). The IL-6, IL-17A and IL-33 levels were higher in the GCF of G4(P-OSA). There was a significant positive correlation between IL-33 in saliva and stage IV periodontitis in G4(P-OSA) (*r*_*s*_ = 0.531). The cytokine profile of the patients in G4(P-OSA) with *Candida* spp. had an increase of the cytokine's levels compared to patients who did not have the yeast.

**Conclusions:**

OSA may increase the risk of developing periodontitis due to increase of IL-1β and IL-6 in saliva and IL-6, IL-17A and IL-33 in GCF that share the activation of the osteoclastogenesis. Those cytokines may be considered as biomarkers of OSA and periodontitis.

## Highlights

• There was a higher prevalence of stage III periodontitis and severe OSA.
• Higher levels of periodontal parameters were correlated with the cytokine's expression.
• Pro-inflammatory cytokines were increased in patients with periodontitis and OSA.
• The IL-33 was correlated to stage IV periodontitis.

## Teaser

**Clinic significance**: Periodontitis and OSA have a bidirectional-relationship: OSA may increase the risk of developing periodontitis, and periodontitis worsens the inflammatory process in OSA.

## 1 Introduction

Periodontitis is a chronic infectious disease caused mainly by strict anaerobic microorganisms [1] that cause an inflammatory response in periodontal tissue, developing periodontal pockets and progressive loss of periodontal insertion and destruction of teeth's supporting structure. These events may be a risk factor for systemic disorders such as cardiovascular disease [2], diabetes [3], rheumatic arthritis [4], and obstructive sleep apnea (OSA) [5,6]. OSA is characterized by the collapse of the upper airway during sleep, resulting in partial or complete obstruction of airflow [7]. OSA is the most common disturbance of sleep condition, with almost 1 billion people affected in the world, and with a prevalence exceeding 50% in some countries, like China, USA, Brazil and India. In Colombia, the prevalence of OSA is approximately 19–26% [8,9].

OSA is associated with a considerably higher risk of cardiovascular illness, such as high blood pressure, coronary heart disease, atrial fibrillation, stroke, diabetes, cancer and death, as well as a severe deterioration in the quality of life and functional capacity [10]. Moreover, OSA has also been associated with periodontitis. Studies in different countries determined that the prevalence of periodontitis is 60–96% in patients with OSA [5,[11], [12], [13], [14]]. Sanders et al. (2015) found that Latin Americans with OSA have a higher risk of severe periodontitis than those without OSA [15]. Latorre et al. (2018) determined the clinical association between periodontitis and OSA in patients with hypertension [16].

The prevalence of periodontitis and OSA may be linked to systemic inflammation. Patients with OSA have higher levels of inflammatory cytokines, adhesion molecules, and activation of circulating neutrophils [17]. The chronic inflammation that underlies OSA might be linked to hypoxia and elevated CO2 levels [18], which could activate transcription factors like NF-κβ, promote the generation of cytokines, reactive oxidant species, and other systemic inflammatory mediators [11,19]. Serum and saliva cytokine levels have been evaluated as candidate biomarkers for the association between OSA and periodontal disease. The association of both conditions might increase the severity of disease by rising the levels of salivary IL-6 [18] and IL-33 [20], changing the composition of biofilm microorganisms, particularly in patients with moderate or severe OSA [21,22]. Il-6 levels in the gingival crevicular fluid (GCF) of OSA patients have not been reported. Other cytokines with pro-osteoclastogenic effects, such as IL-17, may also contribute to the pathogenesis of periodontitis and other diseases [23], but their role in the relationship between periodontitis and OSA has not been investigated. Furthermore, higher IL-1β levels in GCF and similar levels of TNF-α have been demonstrated in patients with OSA and periodontitis [5,18]. Although proinflammatory cytokine concentrations in OSA patients have been documented, some published saliva and serum results are inconsistent. Additionally, there are no studies comparing the levels of cytokines in saliva and GCF in individuals with periodontitis and OSA, to individuals who only have periodontitis or OSA, therefore it is unknown what function IL-17A plays in OSA patients.

Téllez et al. (2022) found that patients with OSA and periodontitis are associated with medical records and that the microorganisms of the orange and red complexes participate in this association [^21^]. The formation of the dysbiotic biofilm was mainly related to the presence of these complexes in association with *Candida* spp., and it could be related with the inflammation as a factor shared between OSA and periodontitis. Further, the pro-inflammatory cytokine levels are increased in serum, saliva and Gingival Crevicular Fluid (GCF) in patients with OSA due to their periodontal condition or indicating a bidirectional relationship between the two conditions, linking periodontitis with OSA. Since both periodontitis and OSA are associated with systemic inflammations possibly involving similar pathways, the objective of this study was to compare the expression of pro-inflammatory cytokines IL-1β, IL-6, IL-17A, IL-33, and TNF-α simultaneously in saliva and GCF in patients with OSA as biomarkers associated with the periodontal condition and its clinical status.

## 2 Materials and methods

### 2.1 Study population and samples

A convenience sample of 84 elegible patients that fulfilled the inclusion criteria (48 women and 36 men; aged between 30 and 71 years) were referred from the Sleep Clinic of the Hospital Universitario San Ignacio and the Sleep Clinic of the Faculty of Dentistry at the Pontificia Universidad Javeriana-PUJ, Bogotá, D.C., Colombia. The present study was carried out following the Declaration of Helsinki of 1975, revised in 2000, and approved by the Research and Ethics Committee of the Faculty of Dentistry (CIEFOUJ No. 005). Following the explanation of their conditions and before to the clinical evaluation, all patients gave written their informed consent. Oral samples (Saliva and GCF) from the patients were collected between May 2019 and March 2021.

The Sleep Clinic of the Hospital Universitario San Ignacio and the Sleep Clinic of the Faculty of Dentistry at the Pontificia Universidad Javeriana-PUJ, Bogotá, D.C., Colombia, referred a convenience sample of 84 eligible patients who fulfilled the inclusion criteria (48 women and 36 men; aged between 30 and 71 years). The current study was conducted in accordance to the Declaration of Helsinki of 1975, revised in 2000 and was given the go-ahead by the Faculty of Dentistry's Research and Ethics Committee (CIEFOUJ No. 005). All patients provided written informed permission following the description of their conditions and before to the clinical evaluation. Patients' oral samples (saliva and GCF) were taken between May 2019 and June 2019.

The following were the inclusion criteria: adults who were 30 years old; who had at least six teeth in their mouth; and had recently undergone a polysomnographic exam (no more than six months before). The following standards for exclusion were stablished: smokers; diabetics; patients who had recently taken antibiotics (in the previous three months); patients who had periodontal treatment previous periodontal treatment (in the last three months); patients who had been treated with continuous positive airway pressure (CPAP) or bilevel positive airway pressure (BPAP); patients who hada pharmacological or surgical treatment for OSA; patients with autoimmune disorders and acute respiratory conditions; and patients who were pregnant.

The presence and severity of OSA in the patients were determined by a polysomnographic study and the Apnea-Hypopnea Index (AHI: mean of apneas or hypopneas per hour). Thus, 5< AHI <15 was considered mild OSA, 15< AHI <30 was considered moderate OSA, and AHI ≥30 was considered severe OSA [24]. For periodontal diagnosis all patients were examined at the Faculty of Dentistry-PUJ through clinic evaluation and panoramic X-rays and classified according to the 2017 World Workshop on the Classification of Periodontal and Peri-Implant Diseases and Conditions [25].

In the dental records the patients' demographic and periodontal parameters were recorded, including age, sex, body mass index (BMI), probing depth (PD), clinical attachment loss (CAL), plaque index (PI), bleeding of probing (BOP), and medical records. According to the severity of OSA and periodontal diagnosis, the patients were classified into four groups: Group 1 (H) healthy patients (n = 23) – G1; Group 2 (P) periodontitis and non-OSA patients (n = 17) – G2; Group 3 (OSA) OSA and non-periodontitis patients (n = 18) – G3; and Group 4 (P-OSA) periodontitis and OSA patients (n = 26) – G4.

### 2.2 Oral samples collection

A 2-mL sample of unstimulated saliva collection was taken and stored at 4 °C. Then samples were centrifuged for 20 min at 4 °C at 10,000 rpm. The supernatant was collected and a protease inhibitor cocktail (Sigma-Aldrich®) was added before being stored at −20 °C until processing. The GCF sample was taken by performing relative isolation of the tooth of interest with gauze and cotton rolls, as well as constant drying with cotton swabs to avoid saliva contamination. The sample was obtained from the deepest pocket in periodontitis patients and from any place in healthy patients by inserting standardized absorbent paper strips (Periopapers®, Oral Flow, Plainview, New York, USA) into the periodontal sulcus for 30 s. Bleeding-contaminated paper strips were discarded. The amount of GCF obtained was measured using Periotron 8010®, an electronic micro-moisture meter instrument (Ora Flow R Inc., New York, USA) (volume measure: periotron units), and the paper strips were immediately placed into 1.5 mL tubes containing 200 μL of sterile phosphate-buffered saline (PBS) solution and a protease inhibitor cocktail (Sigma-Aldrich®). The GCF was vortexed for 10 s before being centrifuged at 2000 rpm for 5 min at 4 °C. The supernatants were then frozen at −20 °C until use [26,27].

### 2.3 Analysis of salivary and GCF cytokines

The levels of IL-1β, IL-6, IL-17A, IL-33, and TNF-α were measured in 50 μL of saliva and GCF using multiplex bead immunoassays (Luminex® Multiplex Assays - Immunoassays, Austin, Texas, USA) according to the manufacturer's instructions (Milliplex MAP Humano TH17, Millipore, Cat. No. HTH17MAG-14 K) and quantification was done in the MAGPIX equipment (Luminex Corporation, Austin, Texas, USA). The standard calibration curve, Software Luminex IS2.3 and Sotfware xPONENT 3.1 (Austin, Texas, USA) were used to determine the concentrations of each cytokine (pg/mL).

### 2.4 Statistical analysis

Data were analyzed with statistical software GraphPad Prism 9.0.2 (GraphPad Software, California, USA), XLSTAT statistical and data analysis solution (Addinsoft, New York, USA) and Software R 4.01 license GPNU (Free Software Foundation, Boston, USA). A descriptive analysis using means, medians, and interquartile ranges, as well as a two-way ANOVA with Tukey's multiple comparisons test, were used to analyze the demographic variables, periodontal parameters of the patients, and cytokine concentrations. The non-parametric Kruskal-Wallis test and the Dunn test for multiple comparisons were used to evaluate the quantitative values of cytokines and the differences among groups (GraphPad Prism 9.0.2). Spearman r rank correlation test was used to determine the correlation between periodontal parameters and cytokine concentrations in saliva and GCF (XLSTAT). All tests were performed with *p*-value <0.05 as significance level.

## 3 Results

### 3.1 Demographic data and clinical periodontal parameters

The demographic variables and periodontal parameters of the study population are displayed in Table 1. There was a higher frequency of men in G4(P-OSA) than in the other groups. The probing depth - PD (mm) was significantly higher in G4(P-OSA) compared with the other groups (*p* = 0.0049). Furthermore, sites (%) PD ≥ 4 mm and BOP (%) showed statistically significant differences between G2(P) and G4(P-OSA) (*p* < 0.001) vs. G1(H).

**Table 1:** Demographic variables and periodontal parameters of the patients.

Clinical variable	**Group 1 (H) (n=23)**	**Group 2 (P)** **(n=17)**	**Group 3 (OSA) (n=18)**	**Group 4 (P-OSA) (n=32)**
Age (years)	45 ± 13	41 ± 10	51 ± 13	50 ± 11
Sex (Females/Males) (n)	16/7	10/7	12/6	11/21
Teeth present	26 ± 4	27 ± 2	24 ± 6	26 ± 6
PD (mm)	1.79 ± 0.47	2.6 ± 0.43	2.57 ± 4.77	13.12 ± 9.71^a^^,^^b^^,^^c^
Sites (%) PD ≥ 4 mm	0.27 ± 0.47	15.45 ± 9.94^a^	2.01 ± 0.15^b^	13.12 ± 9.71^a^^,^^c^
CAL (mm)	1.33 ± 0.73	2.10 ± 0.99	1.52 ± 0.95	2.18 ± 1.04
BOP (%)	12.04 ± 11.86	51.31 ± 26.35^a^	25.16 ± 22.78^a^^,^^b^	48.07 ± 25.39^a^^,^^c^
PI	19.93 ± 12.06	45.24 ± 25.95^a^	38.63 ± 21.23^a^	39.25 ± 18.59^a^
BMI (kg/m2)	25.65 ± 3.38	24.86 ± 3.74	26.18 ± 4.54	28.45 ± 4.09

**Values are given as mean ± standard deviation**.

**PD: probing depth; CAL: clinical attachment loss; PI: plaque index; BOP: bleeding of probing; BMI: body mass index.**

**The statistical analysis was performed with Two-way ANOVA. Tukey's multiple comparisons test. *p*<0.05**.

a **Significantly different from Group 1**.

b **Significantly different from Group 2**.

c **Significantly different from Group 3**.

**Fig. 1:**
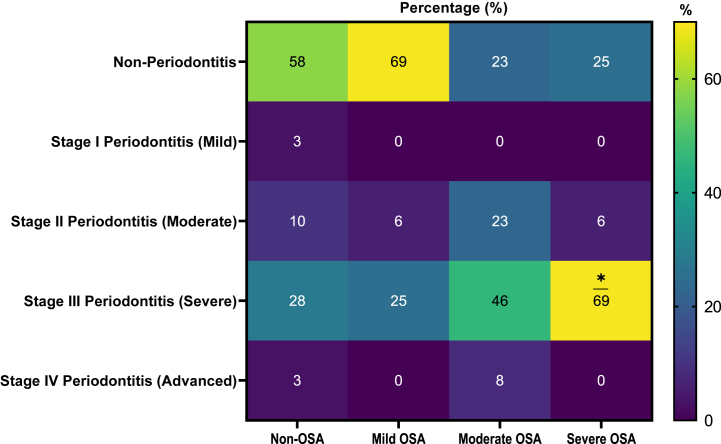
Percentage of the periodontal condition of the patients according to apnea diagnosis; Two-way ANOVA. *p-value = 0.0142.

It was found that 31% of individuals with mild OSA had periodontitis, 77% of individuals with moderate OSA had periodontitis, and 75% with severe OSA had periodontitis. The stage III periodontitis was statistically significant with severe OSA (*p* = 0.0142) (Fig. 1).

### 3.2 Cytokines concentration in saliva and GCF

Salivary IL-1β was the highest of all in G4 (P-OSA) with a slight significance (*p* = 0.057), and there were no significant differences in GCF IL-1β. Salivary IL-6 was significantly highest in G2 (P) (*p* = 0.0044), with differences between G1 (H) (*p* = 0.026) and G3(OSA) (*p* = 0.0168). There was no significant difference with G4 (P-OSA). In addition, salivary TNF-α was highest in G2 (P) (*p* < 0.0001), with differences with G3 (OSA) and G4 (P-OSA) (*p* = 0.0004 and *p* = 0.0009, respectively). In GFC, G3 (OSA) and G4 (P-OSA) had the greatest IL-6 levels; GCF IL-17A was highest in G3 (OSA) patients while GCF IL-33 was elevated in G4 (P-OSA) patients, but with no statistically significant differences. In GCF, patients of G2 (P) and G4 (P-OSA) exhibited similar levels of salivary IL-1β; IL-1β level was higher in G2 (P), followed by G3 (OSA). Similarly, levels of salivary IL-6 were higher in G2(P) and G4(P-OSA), whereas levels of GCF IL-6 were higher in G3 (OSA) and G4 (P-OSA) (Table 2).

**Table 2:** Descriptive statistics of cytokines concentration in saliva and GCF.

Group 1 (H) (n = 23)			Group 2 (P) (n = 17)			Group 3 (OSA) (n = 18)			Group 4 (P-OSA) (n = 26)			*p*-value		
Cytokines (pg/mL)	Sample	Mean	Median	Range	Mean	Median	Range	Mean	Median	Range	Mean	Median	Range	
IL-1β	Saliva	24.28	9.93	0–238.7	36.87	18.46	0–159.2	24.82	12.25	0.39–159.2	52.97	19.45	0–816.6	0.057
	GCF	52.71	30.40	7.1–290.9	72.13	32.1	0–555.8	36.22	32.60	4.9–128.4	83.79	22.10	4.8–568.3	0.84
IL-6	Saliva	1.17	0.00	0–7.98	3.85	**2.78**^a^^,^^b^	0–15.82	3.81	0.00	0–29.72	1.60	1.35	0–7.21	**0.0044**
	GCF	20.2	17.2	6.49–55.93	15.44	11.72	6.13–33.07	26.9	24.49	6.53–73.83	26.54	24.45	6.59–53.19	0.156
IL-17A	Saliva	–	–	–	–	–	–	–	–	–	–	–	–	–
	GCF	35.22	27.99	14.82–110.7	28.44	22.41	11.77–69.53	42.39	32.31	11.86–149.3	36.89	27.06	13.72–92.03	0.1128
IL-33	Saliva	–	–	–	–	–	–	–	–	–	–	–	–	–
	GCF	29.85	26.18	9.47–81.96	21.77	15.53	9.95–47.91	39.93	36.4	10.06–114	37.56	37.71	10.16–73.07	0.071
TNF-a	Saliva	1.42	1.23	0.59–2.65	1.37	**1.47**^b^^,^^c^	0.57–2.12	1.19	0.54	0–6.57	0.84	0.26	0–5.45	**<0.0001**
	GCF	19.09	14.36	0–74.33	11.46	9.03	0–45.40	21.52	12.60	0–98.11	16.56	5.98	0–64.11	0.3957

The statistical analysis was performed with the Kruskal-Wallis test and *post hoc* test: Multiple comparisons using Dunn's multiple comparisons test. The median concentrations per cytokine were compared between groups.

† Significantly different from Group 2.

a Significantly different from Group 1.

b Significantly different from Group 3.

c Significantly different from Group 4.

**Fig. 2:**
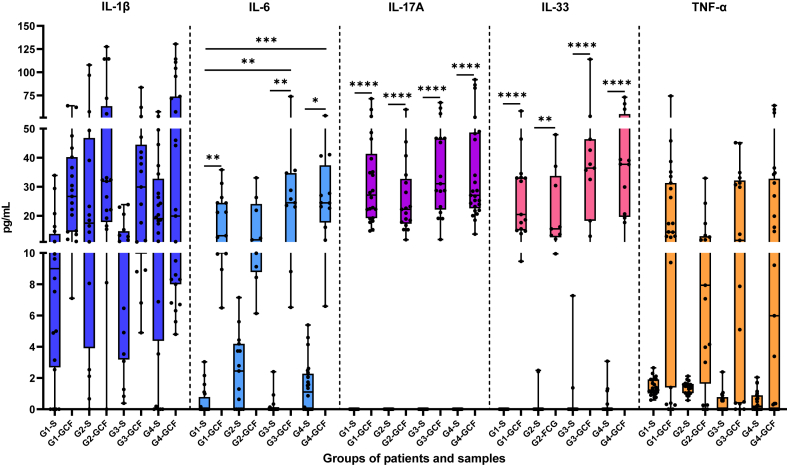
Salivary and GCF concentrations of cytokines in the study groups and samples. Box and whiskers plots represent the median and minimum–maximum values, horizontal bars indicate the significant differences between groups. G1: Group 1 (H); G2: Group 2 (P); G3: Group 3 (OSA); G4: Group 4 (P-OSA). Saliva: S; Gingival Crevicular Fluid: GCF. Kruskal-Wallis test with Dunn's multiple comparisons test: * *p*-value <0.05; ** *p*-value <0.01; *** *p*-value <0.001; **** *p*-value <0.0001.

No concentrations of IL-17A were detected in saliva; however, GCF IL-17A levels were higher in G3 (OSA) and G4 (P-OSA). Salivary IL-33 levels were lower in all groups of patients, but G4 (P-OSA), followed by G3 (OSA) exhibited higher levels in GCF. Similarly, salivary TNF-α levels were lower in all groups of patients; however, G1 (H) and G3 (OSA) exhibited higher TNF-α levels, whereas G2 (P) and G4 (P-OSA) exhibited lower levels. There was a statistically significant difference in IL-6 and IL-33 concentrations between GCF and salivary cytokines being more evident in GCF (Fig. 2).

### 3.3 The tendency of the cytokine concentrations in each group of patients

The percentile plots indicate the tendency of the cytokine concentrations (pg/mL) in each group of patients evaluated. Higher salivary concentrations of IL-1β, IL-6, and TNF-α (62, 5, and 1.4 pg/mL, respectively) were detected in 80% of patients of G2 (P).

**Fig. 3:**
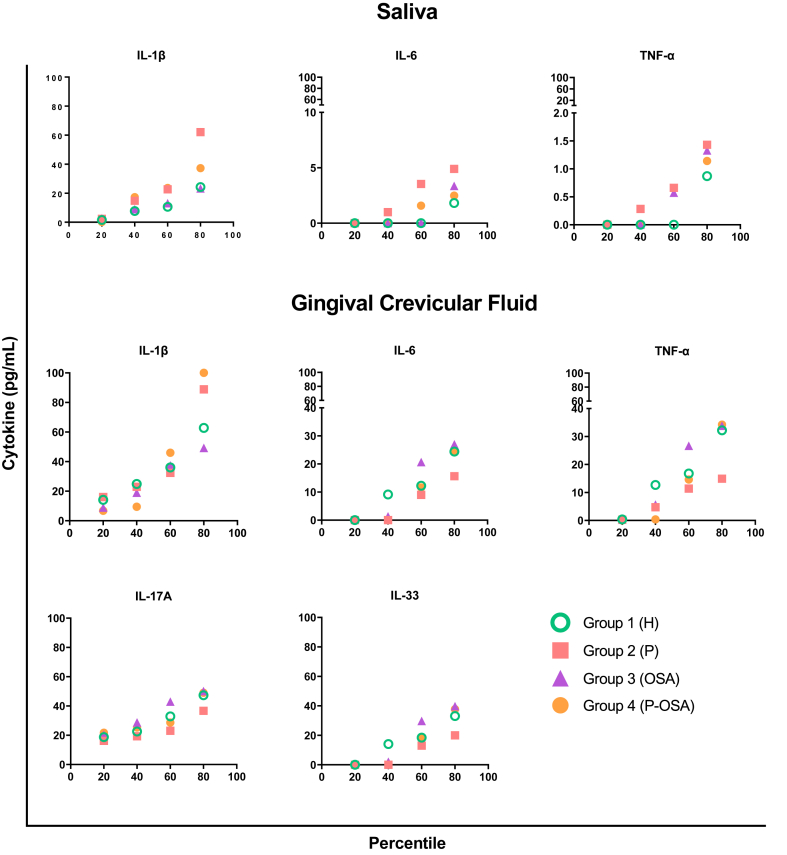
Percentile graph of the cytokine concentrations in saliva and GCF in each group of patients evaluated. Each point represents the cytokine concentration in all patients of each group. The x-axis represents the percentiles and the y-axis the cytokine concentrations in pg/mL. (For interpretation of the references to colour in this figure legend, the reader is referred to the Web version of this article.)

Regarding GCF, 80% of patients of G4 (P-OSA) showed the highest concentration of IL-1β (>100 pg/mL). Also, highest concentrations of IL-6 (27 pg/mL), IL-17A (50 pg/mL) and IL-33 (40 pg/mL) were detected in 80% of patients of G3 (OSA), followed by G4 (P-OSA): IL-6 (27 pg/mL), IL-17A (49 pg/mL) and IL-33 (38 pg/mL). A higher concentration of TNF-α was detected in 80% of patients of G3 (OSA) and G4 (P-OSA) (34 pg/mL) (Fig. 3).

### 3.4 Correlations of periodontal parameters and cytokine concentrations in saliva and GCF

There were significant positive correlations of periodontal parameters and cytokine concentrations in saliva between PI and IL-6 in G3 (OSA) (*rs* = 0.675), PD and TNF-α in G1 (H) (*r*_*s*_ = 0.572), and a significant negative correlation between BOP (%) and TNF-α in G3 (OSA) (*r*_*s*_ = -0.543). Significant negative correlations between periodontal parameters and cytokine concentrations in GCF were found between PI and IL-6 (*r*_*s*_ = −0.549) and PI and IL-33 in G1 (H) (*r*_*s*_ = −0.556) and between all periodontal parameters and IL-6, IL-17A, IL-33 and TNF-α in G2 (P). Positive correlations between IL-1β and all the periodontal parameters were found only in G2 (P), whereas positive correlations between IL-6, IL -17A, IL-33, TNF-α and periodontal parameters PD and BOP (%) were found in G3 (OSA) and G4 (P-OSA) in GCF and saliva (Table 3). Furthermore, Fig. S1 shows graphically the linear positive association (*p* < 0.05), how cytokines change in saliva and GCF in each group of patients, and how they correlate with each other (Fig. S1).

**Table 3:**
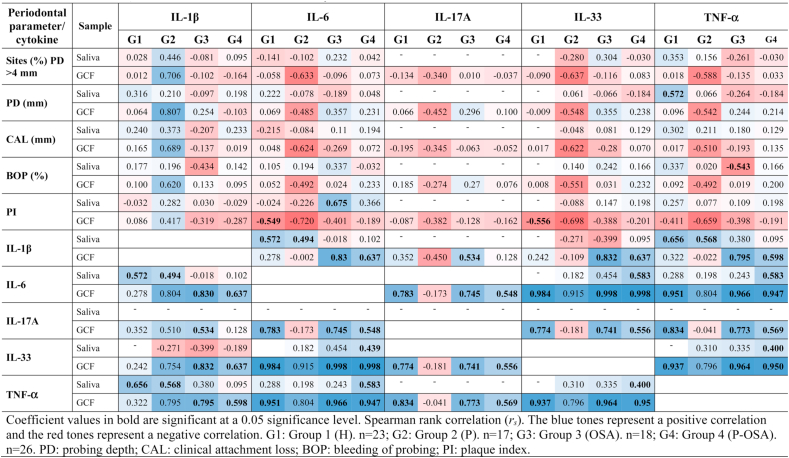
Correlations (*r*_*s*_) of periodontal parameters and cytokines concentrations.

## 4 Cytokines levels in saliva and GCF, periodontal disease stage and severity of OSA

Periodontal disease stages and severity of OSA were related with the cytokines levels in G2 (P), G3 (OSA) and G4 (P-OSA) (Table 4). There was a significant positive correlation between stage IV periodontitis and salivary IL-33 (*r*_*s*_ = 0.531) in G4 (P-OSA), and there was a significant negative correlation between stage II periodontitis and GCF IL-1β (*r*_*s*_ = −0.510, *p* < 0.05) in G2 (P). Moreover, there were only significant positive correlations between mild OSA and IL-1β (*r*_*s*_ = 0.516), IL-6 (*r*_*s*_ = 0.487), IL-33 (*r*_*s*_ = 0.487), and TNF-α (*r*_*s*_ = 0.539) in GCF of G3 (OSA).

**Table 4:**
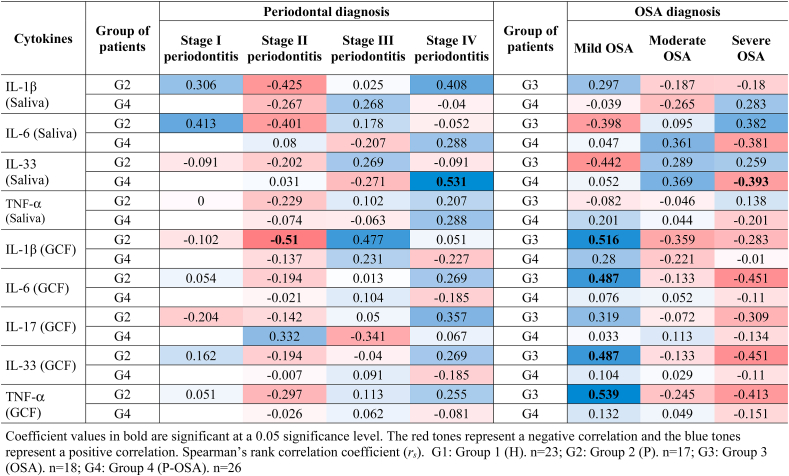
Correlations (r_s_) between cytokines levels in saliva and GCF vs. periodontal disease status and grade of OSA.

### 4.1 Concentrations of cytokines and presence of *Candida* spp. in oral microbiota

**Fig. 4:**
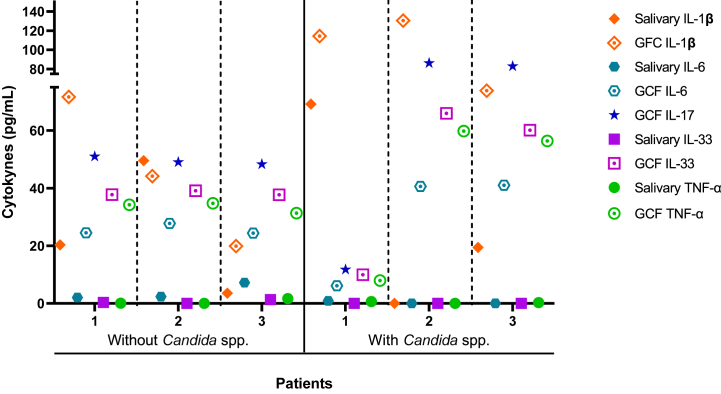
Cytokine concentrations in saliva and GCF in three patients without *Candida* spp. and three patients with *Candida* spp. These patients were classified in the G4 (P-OSA).

Based on the microbiological profiles of six G4 (P-OSA) patients, three of them positive for *Candida* spp. and three of them negative [21], the cytokine concentrations profile showed that the patients with *Candida* spp. had higher cytokine levels relative to those patients who did not have the yeast. In particular, all cytokines concentrations in GCF were the highest (>40 pg/mL), and only IL-1β in saliva was higher in one patient (>60 pg/mL). Regarding to the patients without *Candida* spp., the cytokine concentration profiles were similar in the three patients, with higher levels of cytokines in GCF (20–50 pg/mL) (Fig. 4).

## 5 Discussion

To elucidate the association between OSA and periodontitis, this study compared the salivary and gingival crevicular fluid (GCF) concentrations of five pro-inflammatory cytokines (IL-1β, IL-6, IL-17A, IL-33, and TNF-α) in patients with and without OSA and their association with periodontal condition. The present study demonstrated a high prevalence of stage III periodontitis in patients with severe OSA (69%, *p* = 0.0142). This result is comparable to what Gamsiz-Isik et al. reported (2017) [5], who demonstrated a higher prevalence of severe periodontitis in patients with OSA (48.2%, *p* < 0.001), with moderate-to-severe OSA being more prevalent (52.2%), similarly to results reported by Seo et al. (2013) [12] who determined that 60% of patients with periodontitis had OSA, and Stazić et al. (2022) [28] who concluded that patients with moderate-to-severe OSA had more severe stages of periodontitis (stages III and IV) (*p* = 0.043).

Moreover, in the present study, PD (>4 mm) and BOP (%) were significantly increased in patients with OSA (G3) compared to healthy patients (G1), as reported by Seo et al. (2013) [12] who determined that PD (≥4 mm; *p* < 0.005) and CAL (≥6 mm; *p* < 0.008) were significantly higher in patients with OSA. This evidence suggests that the OSA condition and its physiopathology might be a risk factor for the progression of periodontitis.

Furthermore, different studies have evaluated the immunological relationship between periodontitis and OSA, determining the levels of pro-inflammatory cytokines in saliva, GCF and serum [5,18,20]. As well as the present study, Nizam et al. (2014) reported no statistically significant difference in salivary IL-1β concentrations between a group of healthy patients and a group of patients with OSA [20]. However, our study found high concentrations of IL-1β in saliva (*p* = 0.057) from G2 (P) and G4 (P-OSA) compared to G1 (H) and found that 80% of patients of both groups had the highest concentration of IL-1β (40–60 pg/mL), a determinant cytokine in periodontitis. Although there were no significant differences between the concentrations of IL-1β from G2 (P) and G4 (P-OSA), there was a high level of this cytokine in patients with periodontitis and OSA (G4, P-OSA), suggesting a relationship between OSA and periodontitis.

Similarly, the salivary IL-6 was highest in G2 (P) and G4 (P-OSA), as reported by Nizam et al. (2014, 2016) in patients with OSA, and its concentration was statistically significant in G2 (P) [18,20]. On the other hand, a positive correlation was observed between salivary IL-6 and PI (*rs* = 0.675; *p* < 0.05) in G3 (OSA), and BOP (%) and PI in G4 (P-OSA), which may be explained by the existence of a biofilm in these individuals that increases IL-6 levels as well as the risk of inducing a local inflammatory response; thus, IL-6 could serve as an early salivary biomarker of periodontitis associated to OSA.

Otherwise, the IL-6 in GCF was higher in patients of the G3 (OSA) and G4 (P-OSA) and positively correlated with PD in G3 (OSA) and with PD and BOP (%) in G4 (P-OSA), in comparison with G2 (P) which had lower levels and negative correlations with all periodontal parameters. Our finding of a high concentration of IL-6 in GCF from patients with OSA and periodontitis has not been reported before in literature. Regarding IL-1β in GCF, a possible explanation for the local inflammation is that dryness of mouth caused by OSA, leads to a bacterial colonization as reported by Gamsiz-Isik et al. (2017) [5].

In addition, the present study reports for the first time that concentrations of IL-17A in GCF were high in patients with OSA (G3) and (P-OSA) (G4), with a positive correlation in PD (mm) and BOP (%) in both groups. The IL-17A is secreted by resident memory T helper 17 cells (TH17 cells), which have a homeostatic oral accumulation in presence of IL-6. However, the periodontitis-associated TH17 cells proliferation requires of IL-6 and IL-23 [29] to increase the production of IL-17A. This cytokine has potent osteoclastogenic and inflammatory bone destruction properties due to its capacity to stimulate the expression of RANKL in osteoblasts because it mediates the destruction of connective tissue by inducing the expression of matrix metalloproteinases −3, −9 and −13 (MMP-3, 9, 13) in fibroblasts [30].

Furthermore, there are studies that have shown a significant increase of IL-23 levels in serum of patients with OSA, and its positive correlation with AHI and C-reactive protein (CRP) [31,32], suggesting that high levels of IL-23 in patients with OSA can stimulate the production of pro-inflammatory cytokines by TH17 cells, like IL-17A, which is involved in the development of periodontitis [29].

The present study found lower concentrations of IL-33 in saliva in all groups of patients, while Nizam et al. (2014) found that the concentrations of IL-33 in saliva were higher in patients with OSA, regardless of the severity of the OSA [20]. However, the levels of IL-33 in GCF in the present study were higher in patients from G3 (OSA) and G4 (P-OSA) with positive correlations with periodontal parameters in PD in G3 (OSA) and PD and BOP (%) in G4 (P-OSA). In periodontitis, the expression of IL-33 in gingival epithelial and connective tissue cells acts as an alarmin of tissue damage for the immune system, inducing RANKL expression and triggering the recruitment of RANKL-expressing B and T cells [33,34]. Sozer et al. (2018) reported serum IL-33 concentrations significantly higher in patients with OSA, and hypothesized that OSA influences the levels of IL-33 and is involved in the systemic inflammation produced in OSA [35]. Nizam et al. (2014) have also shown high levels of IL-33 in saliva of patients with OSA [20]. These findings may help explain the bidirectional relationship between periodontitis and OSA, which results from their comorbidity, where the presence of one may cause the other to become more inflammatory. Both conditions increase the presence of IL-17A and IL-33 that share the activation of osteoclastogenesis mediated by RANKL/OPG axis that cause periodontal bone resorption [36,37].

Interestingly, TNF-α plays both anti-inflammatory and pro-inflammatory roles in inflammation: initiating a strong inflammatory response and regulating the intensity and duration of inflammatory processes, respectively. This cytokine plays a variety of roles in the development of periodontitis, including attracting cells to sites of tissue damage and inflammation, and promoting the breakdown of extracellular matrix and bone resorption by increasing the production of IL-1β, IL-6, collagenases, MMPs, and RANKL in gingival epithelial cells [33]. TNF-α levels in saliva and serum were not significantly different between patients with periodontitis and healthy patients; in fact, TNF-α levels were lower in periodontitis patients.

Regarding the levels of TNF-α in OSA, earlier investigations found that both OSA patients and healthy individuals had the same amounts of TNF-α in GCF [5], saliva [18], and plasma [32]. In contrast, other research found a strong relationship between TNF-α and indicators of the severity of OSA and oxygen desaturation [38] among recently diagnosed OSA patients [39]. According to the current findings, with the exception of individuals who only had periodontitis (G2), the patients with OSA and periodontitis and OSA had similarly low levels of TNF-α in saliva. In contrast, the levels of TNF-α in GCF were higher in healthy patients in comparison to patients with OSA and with both conditions. Given the dual functions of TNF-α as an inflammatory mediator, these findings show that there is still a discrepancy in the determination of the levels of TNF-α in patients with periodontitis associated with OSA. This discrepancy may be explained by variations in the demographic and clinical characteristics of the individuals. Therefore, further investigations are required to establish a link between TNF-α levels in saliva, GCF, serum and periodontal condition in OSA assessed simultaneously in the same patients.

According to the correlations of periodontal parameters and cytokine concentrations in saliva and GCF, there was a positive correlation between PD and salivary TNF-α in G1 (H) (*rs* = 0.572; *p* < 0.05). TNF-α may be acting as an anti-inflammatory cytokine in the homeostatic process in healthy individuals.

The anti-inflammatory function of TNF-α in patients without periodontitis may also explain the negative correlation between BOP (%) and TNF-α in G3 (OSA) (*rs* = −0.543; *p* < 0.05). Furthermore, there was a negative correlation between PI and GCF IL-6 (*rs* = −0.549; *p* < 0.05) and between PI and GCF IL-33 in G1 (H) (*rs* = −0.556; *p* < 0.05). These findings might support the homeostatic function of IL-6, although it is unclear whether IL-33 works on the same principle in healthy individuals. Nevertheless, it is important to emphasize the positive correlation between levels of IL-33 and the other cytokines analyzed in patients with OSA and individuals who also have periodontitis, and in the same way, the positive correlation between salivary IL-33 with stage IV periodontitis and moderate OSA in G4 (P-OSA). Further research is necessary to support the role of IL-33 in the physiopathology of OSA in patients with periodontitis.

Despite some authors still concluding that there is little evidence of a possible relationship between periodontitis and OSA, both cause-and-effect and their pathophysiological mechanisms [40], other studies have proposed different mechanisms involving genetic, microbiological, and immunological factors as possible cause-and-effect relationships between OSA and periodontitis [6,18,21,22,41,42]. Both conditions, periodontitis and OSA, may be related by systemic inflammation. Patients with OSA exhibit an increased proliferative potential of natural killer (NK) and CD4 T cells and a decreased capacity of neutrophils to phagocytose bacteria and produce ROS [43,44]. Even the levels of the other enzymes released by neutrophils during inflammation, such as matrix metalloproteinases (MMPs), are significantly lower in patients with severe OSA [44]. Hypoxia and hypercapnia present in patients with OSA have been shown to be related to the apoptosis of periodontal cells [45], that may increase the production of pro-inflammatory cytokines and stimulate transcription factors such as the nuclear transcription factor (NF-κβ) [46]; this factor is involved in the periodontal bone loss [47,48] mediated by the increase of RANKL/OPG (Receptor activator of NF-κβligand/Osteoprotegerin) axis ratio that activates the osteoclastogenic process [37].

Additionally, the presence of *Candida* spp. in the oral microbiota of patients with periodontitis and OSA [21] might well be responsible for the high levels of IL-17A and IL-33 in GCF, worsening periodontal disease. Previous studies have reported the role of IL-17A and IL-33 in response to systemic *Candida albicans* infections, promoting antifungal immunity [49,50]; however, other authors have described the pro-inflammatory function of these cytokines, demonstrating how IL-33 stimulates the production of other cytokines like IL-1 and IL-6 [51], and how the presence of a dysbiotic microbiota causes an increase in IL-17A, which in turn triggers the osteoclastogenic process [52].

In spite of using sensitive multiplex bead immunoassays, the present study faced a limitation in determining values smaller than 5 pg/mL of IL-17A and IL-33 in saliva. Also, comparisons between the two sexes are not possible using the data from this study; hence it is advised to do additional research in the future with a larger sample size and age- and sex-stratified analyses. Analyses based on oxygen saturation and microarousals based on the severity of OSA should also be included. However, the findings of the present study suggest that periodontitis and OSA have a bidirectional-relationship: OSA may increase the risk of developing periodontitis, and periodontitis worsens the inflammatory process in OSA. The pro-inflammatory cytokines IL-6, IL-33 and IL-17A can be proposed as biomarkers of both conditions in GCF due to their ability to trigger the activation of osteoclastogenesis in periodontitis (Fig. S2).

## 6 Conclusions

The present study suggests a bidirectional and comorbid link between OSA and periodontitis. There was a higher prevalence of stage III periodontitis and severe OSA, as well as higher levels of periodontal parameters (PI and BOP), and expression of IL-1β and IL-6 in saliva and IL-6, IL-17A, and IL-33 in GCF, in patients with periodontitis and OSA. These cytokines may be considered biomarkers of both conditions, emphasizing the association between IL-33 and stage IV periodontitis.

Our results suggest that there is a potential diagnosis of association between individuals with OSA and periodontitis; individuals with OSA should get a periodontal screening; and individuals with periodontitis should get a sleep study to determine OSA. Further studies are necessary to analyze the role of pro-inflammatory cytokines in the physiopathology of OSA in patients with periodontitis.

## Declarations

### Author contribution statement

Mayra A. Téllez Corral: Conceived and designed the experiments; Performed the experiments, Analyzed and interpreted the data; Contributed reagents, materials, analysis tools or data; Wrote the paper.Eddy Herrera Daza; Juliana Velosa Porras; María E. Cortés; Liliana Otero; Claudia M. Parra Giraldo; Nelly S. Roa Molina: Analyzed and interpreted the data; Contributed reagents, materials, analysis tools or data.Natalia Arango Jimenez; Darena Z. Morales Vera; Catalina Latorre-Uriza; Francina M. Escobar Arregoces; Patricia Hidalgo Martinez: Contributed reagents,Materials, analysis tools or data.Nelly S. Roa Molina: Conceived and designed the experiments.

### Funding statement

Dr. Mayra Alexandra Téllez Corral was supported by Banco de la República [4.509].

### Data availability statement

Data included in article/supp. material/referenced in article.

### Declaration of interest's statement

The authors declare no competing interests.
