# Supplementary material for: Biomarkers for the severity of periodontal disease in patients with obstructive sleep apnea:IL-1 β, IL-6, IL-17A, and IL-33
Source: Heliyon. 2023 Mar 14;9(3):e14340. doi: 10.1016/j.heliyon.2023.e14340 (PMC10031375; doi:10.1016/j.heliyon.2023.e14340)
Supplement: Multimedia component 1 [file mmc1.docx]

**
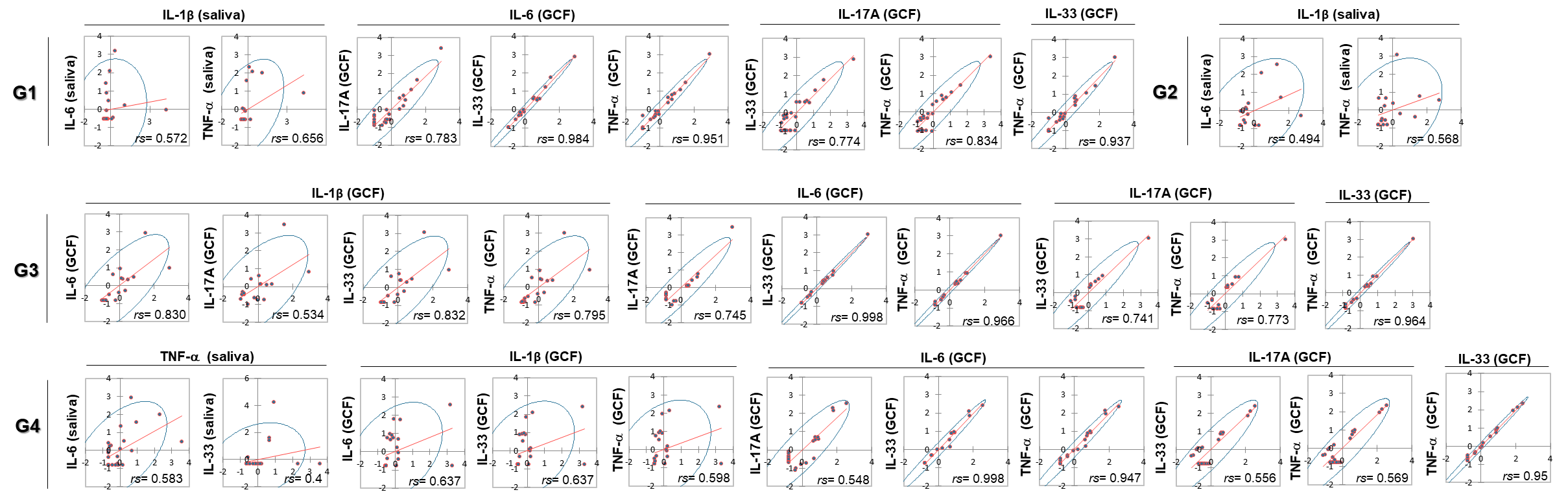
****Figure S1.** Scatter plot matrix of the statistical correlation in saliva and GCF concentrations of each cytokine of each group of patients. Each panel is a scatter plot of one cytokine against another cytokine. The trend lines are presented for each plot along with Spearman’s rank correlation coefficient (*r_s_*) and the red color of the data points in the scatter plots reveals a positive correlation. G1: Group 1 (H); G2: Group 2 (P); G3: Group 3 (OSA); G4: Group 4 (P-OSA). All correlations were statistically significant at a level of *p* < 0.05.


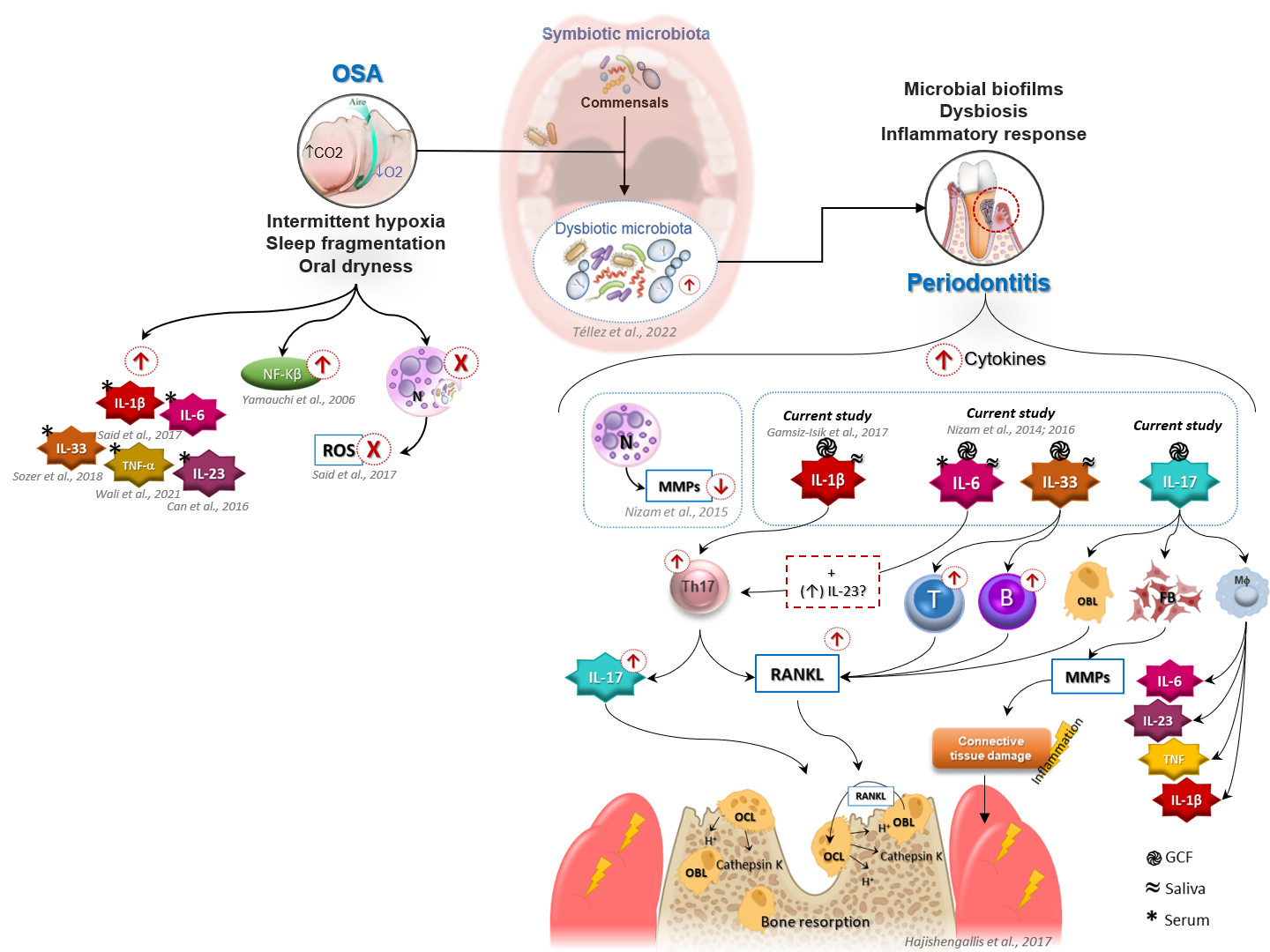


**Figure S2.** A schematic diagram explaining the immunomicrobial physiopathology of periodontitis associated to OSA. In patients with OSA, the local inflammation is due to dryness of mouth driven by OSA, leading to the multi-microorganisms colonization that triggers the activation of different cells of the immune response and stimulate NF-κB favoring the production of cytokines locally in the GCF, among them IL-6, IL-33 and IL-17, that may increase the osteoclastogenesis mediated by RANKL/OPG axis and with it, the progression of the periodontal disease. Salivary IL-1β and IL-6 are a reflection of the systemic inflammation of OSA and periodontal disease indicating a bidirectional relationship. OSA, obstructive sleep apnea; IL, interleukin; TNF-α, Tumour Necrosis Factor-alpha; NF-κB, Nuclear factor kappa beta; N, neutrophil; ROS, reactive oxygen species; MMPs, matrix metalloproteinases; Th17, T-helper type 1 cell; T, T-cell; B, B-cell; OBL, osteoblast; FB, fibroblast; Mφ, macrophage; RANKL, receptor activator of nuclear factor-kappaB ligand; OCL, osteoclast; H+, hydrogen ions.
